# CNN-Based Vehicle Bottom Face Quadrilateral Detection Using Surveillance Cameras for Intelligent Transportation Systems

**DOI:** 10.3390/s23156688

**Published:** 2023-07-26

**Authors:** Gahyun Kim, Ho Gi Jung, Jae Kyu Suhr

**Affiliations:** 1 Department of Intelligent Mechatronics Engineering, Sejong University, Seoul 05006, Republic of Korea; 2 Department of Electronic Engineering, Korea National University of Transportation, Chungju-si 27469, Republic of Korea

**Keywords:** vehicle position estimation, bottom face quadrilateral, deep neural network, vehicle-to-infrastructure (V2I), intelligent transportation system, surveillance camera

## Abstract

In intelligent transportation systems, it is essential to estimate the vehicle position accurately. To this end, it is preferred to detect vehicles as a bottom face quadrilateral (BFQ) rather than an axis-aligned bounding box. Although there have been some methods for detecting the vehicle BFQ using vehicle-mounted cameras, few studies have been conducted using surveillance cameras. Therefore, this paper conducts a comparative study on various approaches for detecting the vehicle BFQ in surveillance camera environments. Three approaches were selected for comparison, including corner-based, position/size/angle-based, and line-based. For comparison, this paper suggests a way to implement the vehicle BFQ detectors by simply adding extra heads to one of the most widely used real-time object detectors, YOLO. In experiments, it was shown that the vehicle BFQ can be adequately detected by using the suggested implementation, and the three approaches were quantitatively evaluated, compared, and analyzed.

## 1. Introduction

Surveillance camera-based vehicle detection has been widely used in various intelligent transportation system (ITS) applications [1,2]. In many of these, an accurate vehicle position is required [3,4,5]. For instance, autonomous driving based on vehicle-to-infrastructure (V2I) prevents accidents or control vehicles using accurate positions provided by infrastructure surveillance cameras. Various sensors have been used for vehicle detection, including monocular cameras [6,7,8,9], lidars [10,11], lidar–camera fusion [12,13], and stereo cameras [14]. Among them, monocular cameras are preferable because they can be installed at a low cost and require less computational load. In addition, widely spread existing monocular surveillance cameras can be reused for ITS applications [8]. Considering these advantages, this paper presents a method for accurately estimating the vehicle position using a monocular surveillance camera.

The most representative way to estimate the vehicle position using a monocular camera is as follows. It first detects the vehicle position in-image coordinates and then transforms it to world coordinates using homography, which expresses the relationship between the ground in-image coordinates and in-world coordinates. This approach has a vital assumption that the detected vehicle position in-image coordinates should be on the ground surface. If this assumption is not satisfied, the vehicle position accuracy in-world coordinates is degraded. Therefore, vehicle positions in-image coordinates should be carefully detected when using monocular cameras.

**Figure 1:**
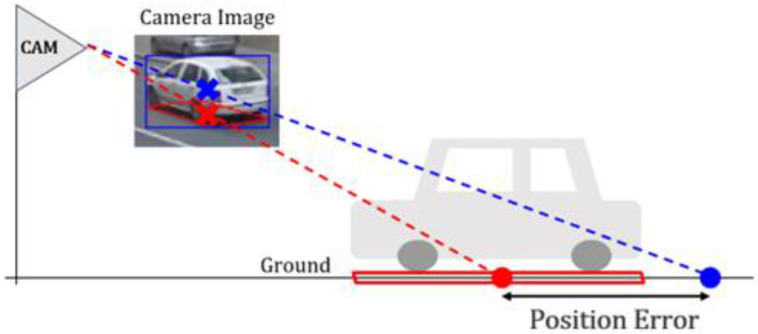
Vehicle position error when using the center of the bounding box. The blue cross is the center of the blue bounding box. The red quadrilateral and cross are the bottom face of the vehicle and its center. The distance between the blue and red dots on the ground indicates the position error.

The simplest method for finding the vehicle position is using the center of the bounding box given by object detectors [15,16,17]. This method is computationally efficient and easy to implement because it simply reuses conventional object detection results. However, this method does not follow the aforementioned assumption because the center of the bounding box is not located on the ground surface. Thus, it produces erroneous positioning results, as shown in Figure 1. In this figure, the blue cross in the image indicates the center of the bounding box. If the blue cross is used as the vehicle position in-image coordinates, the vehicle position in-world coordinates are calculated as the blue dot on the ground. This blue dot includes an error because the correct vehicle position on the ground is the red dot on the ground. The amount of this position error depends on the relative position between the vehicle and camera in-world coordinates. To reduce this vehicle position error, the red cross should be detected in the image because it corresponds to the correct vehicle position on the ground (red dot) in Figure 1 [18]. The red cross indicates the bottom face quadrilateral (BFQ) center of the vehicle in the image.

**Figure 2:**
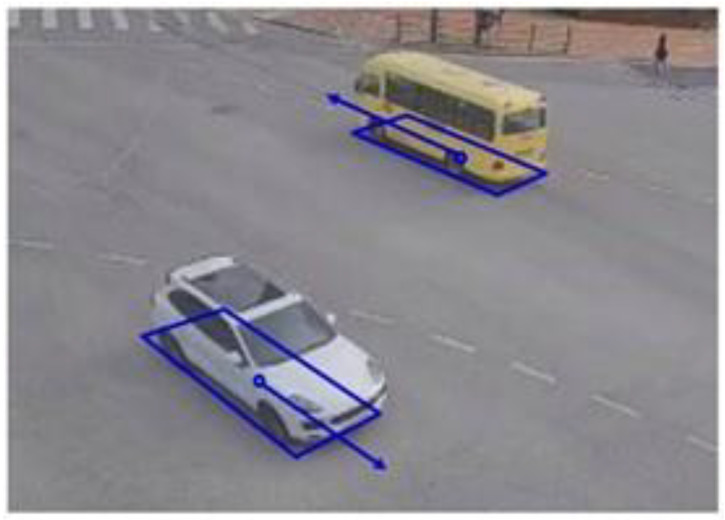
The usefulness of the vehicle BFQ. Quadrilaterals, circles, and arrows indicate BFQs, their centers, and their directions, respectively.

Therefore, this paper focuses on detecting the BFQ to enhance vehicle position accuracy in visual surveillance environments. Although there have been some methods for detecting the vehicle BFQ using vehicle-mounted cameras, few studies have been con-ducted using surveillance cameras. For vehicle position, it is enough to estimate only the single point, the BFQ center, but the BFQ with four corners is preferred because it gives additional useful information, such as the size and direction of the vehicle, as shown in Figure 2.

The proposed vehicle BFQ detection and the 3D vehicle detection both provide information on the vehicle’s location, direction, and size. Compared to BFQ detection, the only additional information provided by the 3D vehicle detection is the vehicle’s height. The vehicle’s height is rarely used in V2I-based autonomous driving since this application only requires the vehicle’s location, direction, and size. For the 3D vehicle detection, four points of the vehicle top face quadrilateral (TFQ) should be estimated in addition to four points of the BFQ. Therefore, the 3D vehicle detection has the following drawbacks compared to the proposed vehicle BFQ detection: (1) it requires more effort for labeling to build the dataset, (2) it increases the computational cost and network size, and (3) it degrades the detection performance because the TFQ’s shape varies a lot and does not fit the vehicle size, unlike the BFQ.

Single image-based 3D reconstruction can also be used for vehicle positioning in surveillance environments. The most representative approach for this is monocular depth estimation, which is the task of estimating the depth value of each pixel given a single RGB image [19]. This approach has the advantage of obtaining dense distance information with a single image but has the following disadvantages in surveillance camera situations. First, this approach has difficulties in handling various backgrounds and objects that appear in the real world because of the limited dense depth labels. This disadvantage is more prominent in surveillance situations because the camera’s intrinsic properties (field of view, distortion, etc.) and extrinsic properties (installation height, angle, etc.) are very diverse. Second, this approach has limitations in handling distant objects captured with small sizes in surveillance camera images. The positions of distant objects are difficult to estimate based on monocular depth estimation because it often ignores small-sized objects or inaccurately estimates their depths. Last, this approach uses a large network with a high computational cost. Since surveillance cameras should use low-end processors when considering their reasonable prices, using a small network with fast inference time is preferable.

Recently, there has been an increase in the convolutional neural network (CNN)-based methods for detecting the BFQ using a monocular camera. Based on the way to represent the quadrilateral, these methods can be categorized into three approaches: corner-based, position/size/angle (PSA)-based, and line-based. The corner-based approach represents the BFQ with its four corners [6,7,8,9,13,20,21,22,23,24,25,26]. The PSA-based approach represents the BFQ with its position, size, and angle [27,28,29,30,31,32,33,34,35,36,37]. The line-based approach represents the BFQ with four lines that compose its edge [3,38,39,40,41]; further details will be explained in the next section. Since most of these methods are for vehicle-mounted cameras, their effectiveness in surveillance environments has not been well addressed. Therefore, this paper conducts a comparative study on various approaches for detecting the vehicle BFQ in surveillance environments.

The proposed method has two main contributions. First, it presents a guideline for developing the vehicle BFQ detection method using a monocular surveillance camera by quantitively evaluating various approaches. Second, it suggests a way to implement the vehicle BFQ detectors by adding extra heads to one of the most widely used real-time object detectors, YOLO. This enables researchers to develop their own real-time vehicle BFQ detectors more easily by slightly changing their current object detectors. This paper explores all three aforementioned approaches: corner-based, PSA-based, and line-based. The experimental results show that the corner-based approach is the most effective for vehicle BFQ detection. This is because, in this approach, position errors of four corners have less effect upon the shape of the vehicle BFQ. In contrast, in the other two approaches, position errors of PSAs and lines highly degrade the BFQ detection performance.

The rest of this paper is organized as follows. Section 2 reviews previous studies related to vehicle BFQ detection. Section 3 explains the implementation details of various BFQ detection approaches. Section 4 describes the experimental results and discussions. Finally, this paper is concluded with a summary in Section 5.

## 2. Related Works

**Figure 3:**
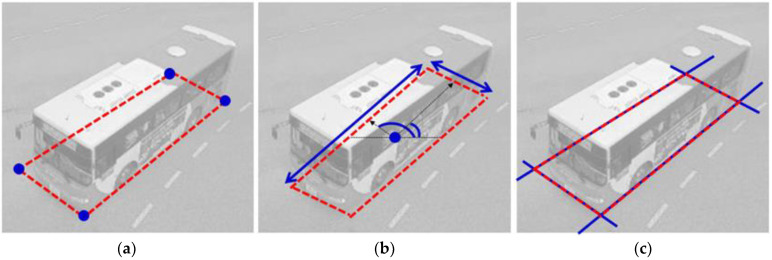
Three approaches for vehicle BFQ detection. The red quadrilaterals represent the vehicle BFQs. (**a**) The corner-based approach detects the BFQ by finding four corners (blue dots); (**b**) the PSA-based approach detects the BFQ by finding the position (blue dot), size (blue arrows), and rotation angle (blue arcs); and (**c**) the line-based approach detects the BFQ by finding four quadrilateral lines (blue lines).

Vehicle detection methods can be categorized into four approaches according to the complexity of expressing the vehicle: bounding box-based [42], bottom face quadrilateral (BFQ)-based [5], 3D bounding box-based [33], and wire frame-based [43]. Since this paper deals with the BFQ-based approach that provides the vehicle’s position, size, and direction while requiring less computation, the detailed related literature has been reviewed only for this approach. The methods in the BFQ-based approaches can be classified into three methods: corner-based, position/size/angle (PSA)-based, and line-based. Figure 3 shows brief descriptions of these three approaches. The BFQ, which is the detection target, is shown in red. The blue features represent what each approach finds to detect the BFQ. All three approaches detect the BFQ by finding the four corners, the position/size/angle, and the four lines of the BFQ, respectively.

### 2.1. Corner-Based Approach

The corner-based approach detects the vehicle BFQ by finding the corners of the bounding box, which can be a rectangle or cuboid. The detected corners are used directly or auxiliary to generate the corresponding rectangle or cuboid. In [20,23], eight corners were detected directly by regressing offsets from the reference point to each corner. The reference point was the center of the 2D bounding box, and the offsets were the coordinate differences along the image axes, which are the x and y axes, from the reference point to the corner. The offsets were encoded as ratios of the width and height of the 2D bounding box, respectively. On the other hand, [22] defined the lower left corner of the 2D bounding box as the reference point and encoded the offset as a ratio to the width of the 2D bounding box. In [21], it also directly detects eight corners. They designed a network consisting of four subnetworks of 2D detection, instance depth estimation, projected 3D center estimation, and local corner regression. The eight corners were predicted in local coordinates with the center of the 3D bounding box as the origin. Inspired by CenterNet, [6,24,44] created heatmaps to determine the eight corners and the center of the 3D bounding box. In particular, [6] regressed offsets from the main center to each corner. The main center is the 2D bounding box center. In [9], the corners were located by transforming the ROI from the 2D bounding box to column vectors and passed it through the Softmax layer. In [7,13,26], only four out of eight corners were detected. In addition to four corners of the BFQ, [13,26] found two heights and one height information from the ground to the bottom and top face, respectively. In [7], the Single Shot MultiBox Detector [45] was extended to output 3D bounding shapes using predefined four corners instead of 2D bounding boxes. Three of the four corners form the BFQ, and the remaining corner is used to determine the height. Papers [8,25] differ in using a wire model for a vehicle. In [8], key points were detected that are predefined using a 3D computer-aided design (CAD) model. Vehicles are classified into 5 models, and 14 points were designated for each model. The network uses mask R-CNN [46] as the backbone and directly regresses 14 points in one of the 3 sub-networks. In [25], vehicles were classified into four models and each model was assigned a 3D bounding box with a cuboid shape and a 3D shape defined by several points. The points of the 3D shape were regressed using the network.

### 2.2. PSA-Based Appraoch

The PSA-based method detects the vehicle BFQ by finding the position, size, and angle of the bounding box. In [27,28,29,30,31,32,33,34,35,36,37], the position, size, and rotation angle of the bounding box are obtained as the vehicle detection result. In [27], which has two subnetworks, depth-aware convolution was used to extract local features for object detection. The position, size, and angle were estimated by predicting the offset from the anchor. They encoded the offset as a ratio to the width or height of the 2D bounding box for position detection. The size and angle were determined by predicting the residuals and the log-scale offsets from the anchor boxes, which varied in size with the depth. In [31], average sizes were calculated from the dataset and used as anchor boxes. They found the dimension of the vehicle by regressing the residual on the anchor. The angle was decoded into a vectorial representation such as sine and cosine in the network. In [34], the center of the object was found using CenterNet and regressed the log-scale offset on the average size of each class. The angle was regressed on a vectorial representation as in [31]. In [35], the centerness was found, represented with 2D Gaussian distribution, which describes how closer points are to centers and regresses the offset from the center, the size, and the angle. The attributes such as size and angle are regressed from the shared head that consists of convolutional blocks. In [37], they regressed the discretization offset for the position, the log-scale offset for the size, and the vectorial representation for the angle detection. Some methods [28,29,30,32,33,36] use 2D bounding box information for position detection. Among them, [28,30,32,36] used the center of an edge of the 2D bounding box or the center of the bounding box. In [36], they refined the center position by estimating the disparity for more accurate positioning. In [33], it was assumed that the center of the 2D bounding box coincides with the center of the 3D bounding box. These methods have in common that they use the average size of each class for dimension detection. However, the difference in detail is that [28,36] regress the log-scale offset, while the others regress the residual with the mean size. In [29,30,32,36], they found the angle using the multi-bin method presented in [33]. The multi-bin method divides an angle with continuous values into a finite number of bins and regresses the residual from the center in each bin.

### 2.3. Line-Based Approach

The line-based approach detects the vehicle BFQ by finding four lines that form it. In [3,40], they found the vehicle blob by segmentation and then computed the tangent lines of the blob. By calculating the intersection of these lines, they obtained the bounding box. Some other methods trained the network to output parameters of the lines. In [38], they introduced a parameter corresponding to the height of the vehicle. The parameter is a ratio that allows it to calculate the location corresponding to the height of the vehicle on the vertical edge of the bounding box. By finding a straight line connecting the point calculated from the parameters and the vanishing point, the vehicle position can be calculated. In [39,41], they considered that some vertices of the 3D bounding box meet the edge of the 2D bounding box. In [39], they defined a local coordinate system, predicted several parameters for regression and classification, and generated the bounding box; unlike [39,41]. who generated the bounding box based on the camera calibration. In [47], 3D templates for each class were defined and the bounding box was obtained by using the similarity between vehicles and templates.

## 3. Implementation Details

This paper implements and compares 11 representative methods for vehicle BFQ detection based on the literature review. These methods are implemented by adding extra heads to one of the most widely used real-time object detectors, YOLO.

### 3.1. Implementation Using YOLO

**Figure 4:**
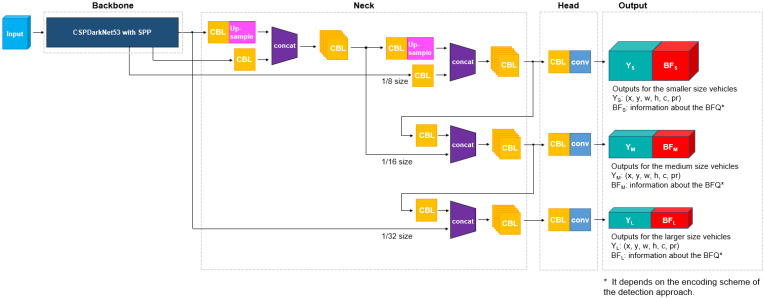
Structure of the proposed vehicle BFQ detector. CBL indicates a combination of convolution, batch normalization, and leaky ReLU. (x, y) and (w, h) are the position and size of the bounding boxes, respectively. c and pr are the confidence score and the class probability, respectively.

YOLO is one of the most widely used one-stage detectors used in various fields such as traffic surveillance [48,49], autonomous driving [50,51], unmanned aerial vehicles [52,53], and robotics [54]. Among several YOLO variants, YOLOv4 was selected in this paper because it has been proven for a considerable time in a variety of applications and has demonstrated a compromise between detection accuracy and computational cost in various frameworks [55,56]. The vehicle BFQ detectors are implemented by adding extra heads to YOLOv4, as shown in Figure 4. The extra heads for the BFQ detection are represented as red BF blocks, which are added to the Y blocks for vehicle detection. Except for the BF blocks (red blocks), the structure is the same as that of YOLOv4, which consists of the backbone, the neck, and the head. The backbone is CSPDarkNet53 [57] with a spatial pyramid pooling (SPP) module [58] and trained on the COCO dataset [59]. The neck of the network consists of convolution (C), batch normalization (B), leaky ReLU (L), up-sampling, and concatenation modules. Most layers use the first three modules together, but only the last layer of the head uses a convolution. The combination of the three modules (convolution, batch normalization, and leaky ReLU) is denoted as CBL in Figure 4. The up-sampling and concatenation modules are used twice and four times to create multi-scale feature maps. The network takes a color image as an input and produces three feature maps with different scales as outputs. Y_S_ + BF_S_, Y_M_ + BF_M_, and Y_L_ + BF_L_ denote the three output feature maps in Figure 4. Y and BF include information on bounding boxes and BFQs, respectively, and subscripts S, M, and L indicate their scales. The sizes of the output feature maps are 1/8, 1/16, and 1/32 of the input image size, respectively. The output feature map for the bounding box (Y) is the same as that of YOLOv4, including the position and size of the bounding box, the confidence score, and the class. The output feature map for the BFQ (BF) depends on the encoding scheme of the BFQ detection approach. Details are given in the following sections.

### 3.2. Implementation of Corner-Based Approach

**Figure 5:**
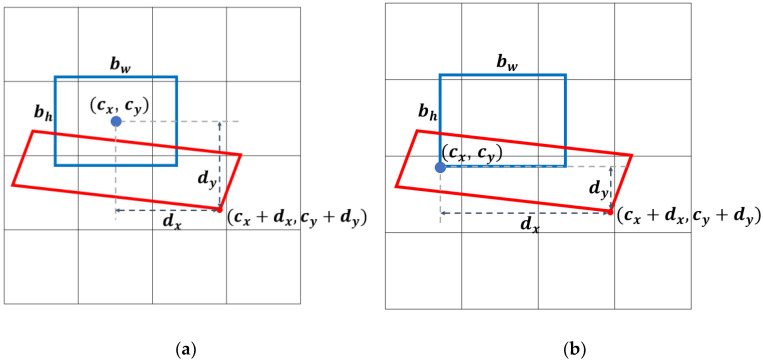
Definition of the origin for the corner-based approach. The blue rectangles are the anchor box with the size $b_{w}\times b_{h}$, and the red quadrilaterals are the vehicle BFQ: (**a**) The origin $\left(c_{x},c_{y}\right)$ is defined as the center of the anchor box; (**b**) The origin $\left(c_{x},c_{y}\right)$ is defined as the lower left corner of the anchor box.

The vehicle BFQ has four corners, and their positions in the image are represented as the sum of an origin coordinate and an offset. Therefore, detecting the corner is equivalent to estimating the offset from the predefined origin to the corner. The origin can be determined in various ways, but in this paper, two methods are applied by referring to the existing methods [20,21,22,23]. The offsets are also encoded in two ways. Thus, the total number of combinations of origin and offset is four. Figure 5a,b are cases where the origin ($c_{x}$,$c_{y}$) is defined differently. In Figure 5, the red quadrilateral represents the vehicle BFQ, and the blue rectangle represents the anchor box of the size $b_{w}\times b_{h}$. The anchor boxes are the same as those in YOLOv4, where nine anchor boxes are used (three for each scale). The anchor boxes were selected by applying k-means clustering to the sizes of the objects in the COCO dataset. Figure 5a is the case where the center of the anchor box is defined as the origin, and Figure 5b is the case where the lower left corner of the anchor box is defined as the origin. As you can see in this figure, a corner coordinate of the BFQ is far from the origin by the offset ($d_{x}$,$d_{y}$). The offsets are encoded in two ways using the anchor box size:

(1) $$d_{x}=\delta_{x}\cdot b_{w}\;,\;\;d_{y}=\delta_{y}\cdot b_{h}$$

(2) $$d_{x}=\delta_{x}+b_{w}\;,\;\;d_{y}=\delta_{y}+b_{h}$$

where $\delta_{x}$ and $\delta_{y}$ are the outputs of the added extra heads. $\delta_{x}$ and $\delta_{y}$ in (1) and (2) are the ratios to the anchor box size and the differences from the anchor box size, respectively. Table 1 shows that the four variations of the corner-based approach are created by the combination of origin definitions and offset encoding methods.

### 3.3. Implementation of PSA-Based Approach

The methods included in the PSA-based approach identify the center, size, and angle of the vehicle for the position detection [27,28,29,30,31,32,33,34,35,36,37]. This approach detects the BFQ center for position detection, the width and height for size detection, and the local and global angles for rotation detection. Here, the local angle indicates how much a rectangle is skewed in order to become a parallelogram, and the global angle indicates how much a parallelogram is rotated. The angles can be used to determine the direction in which the vehicle is moving. Current methods usually establish a reference value, such as the 2D bounding box, the 3D anchor box, or the average value by class, and then detect the offsets from this reference value. In this paper, the reference value is replaced by a 2D anchor box, and the BFQ is assumed to be a parallelogram for simplicity [22,60].

**Figure 6:**
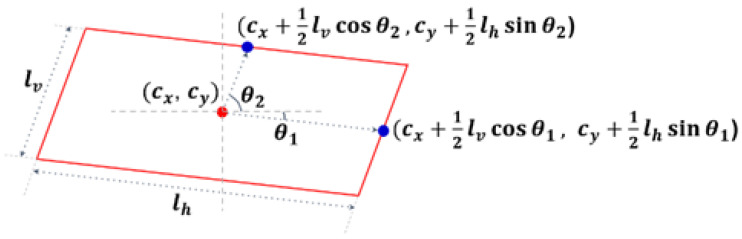
The vehicle BFQ described with its position, size, and angle. The red parallelogram is the vehicle BFQ. The red dot and the blue dot indicate the center of BFQ, and the center of the sides tilted by $\theta_{1}$ and $\theta_{2}$ from the image axes, respectively.

Figure 6 shows a vehicle BFQ represented by the position, size, and angle. In this figure, the red parallelogram of size $l_{v}\times l_{h}$ is the BFQ, and the red dot is its center $\left(c_{x},\;c_{y}\right)$. The center is detected by the C2 method, which is a method of the corner-based approach. The size of the BFQ is equal to the length of the two sides, $l_{v}$ and $l_{h}$, of the parallelogram in Figure 6. The size is encoded using the anchor box size as:

(3) $$l_{v}=r_{x}+b_{w}\;,\;\;l_{h}=r_{y}+b_{h}$$

(4) $$l_{v}=e^{r_{x}}\cdot b_{w}\;,\;\;l_{h}=e^{r_{y}}\cdot b_{h}$$

where $r_{x}$ and $r_{y}$ are the outputs of the added extra heads. $r_{x}$ and $r_{y}$ in (3) and (4) are the differences from the anchor box size and the log-scaled ratios to the anchor box size, respectively.

The angle of the BFQ can be found by using the vectors from the center (red dot) to the centers of the two sides of the parallelogram (blue dots) in Figure 6. Using the BFQ center, the lengths of the sides, and the angles, the vector representations of the centers of the sides are $\left(c_{x}+\frac{1}{2}l_{v}\cos\theta_{1},c_{y}+\frac{1}{2}l_{h}\sin\theta_{1}\right)$ and $\left(c_{x}+\frac{1}{2}l_{v}\cos\theta_{2},c_{y}+\frac{1}{2}l_{h}\sin\theta_{2}\right)$. In this paper, two methods of angle detection are used: direct estimation of the angle θ and estimation of (cos⁡θ,sin⁡θ).

In the PSA-based approach, there is a change in the output dimension of the added extra heads for each variation. The output dimensions of the added extra heads for position and size detection are the same. However, the output for angle detection is $\left(\theta_{1},\;\theta_{2}\right)$ or $\left(\cos\theta_{1},\;\sin\theta_{1},\cos\theta_{2},\;\sin\theta_{2}\right)$. According to different encoding methods of size and angle detection, four variations are created. They are shown in Table 2.

### 3.4. Implementation of Line-Based Approach

The vehicle BFQ can be detected by estimating four straight lines. This paper presents two methods in the line-based approach: line parameter-based and intersection point-based.

#### 3.4.1. Line Parameter-Based Method

**Figure 7:**
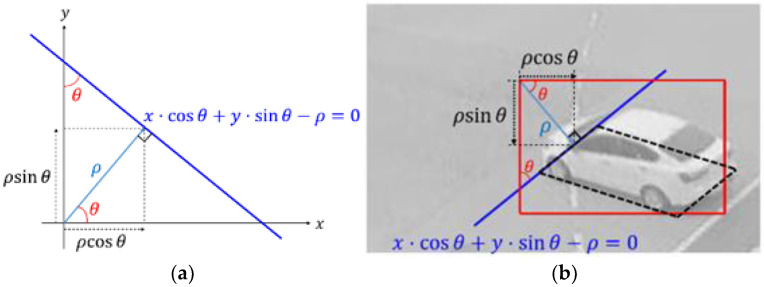
Representation of one side of the BFQ as the normal form of a linear equation: (**a**) The normal form of a linear equation for the blue line that the angle with the *x*-axis is θ and the normal distance from the origin is ρ; (**b**) the representation of the blue line of the black BFQ as (**a**).

A straight line can be expressed by the normal form of a linear equation. As shown in Figure 7a, if the blue line has a positive angle θ with the x-axis and a normal distance ρ from the origin s, its linear equation is x⋅cos⁡θ+y⋅sin⁡θ−ρ=0. In the same way, the four lines of the BFQ can be written as four linear equations. Figure 7b shows how one side of the BFQ is expressed as a linear equation. In this figure, the red rectangle and the black dashed quadrilateral are the bounding box and the BFQ, respectively. The blue line is the extension of one side of the BFQ. If the upper left corner of the bounding box is the same as the origin of the coordinate system, the blue line has the angle θ and the normal distance ρ as the line in Figure 7a. As a result, the equation form of the blue line is also x⋅cos⁡θ+y⋅sin⁡θ−ρ=0. Therefore, the outputs of the added extra heads are four sets of (cos⁡θ,sin⁡θ,ρ) and the vehicle position is finally detected by calculating the intersection of the four straight lines generated from these sets. This method is referred to as L1 in the following.

#### 3.4.2. Intersection Point-Based Method

**Figure 8:**
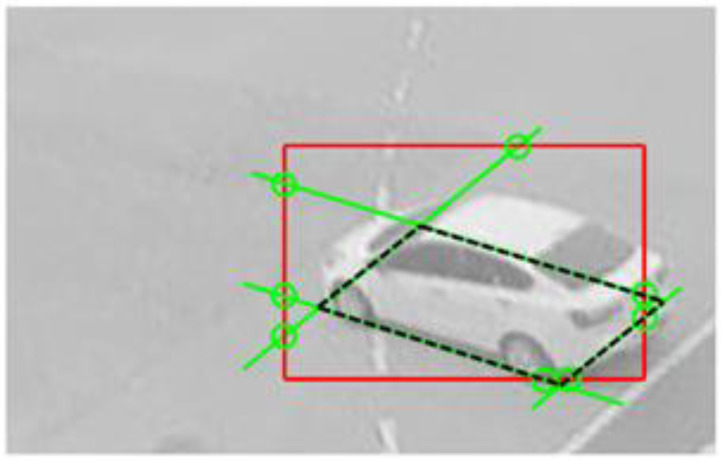
Intersection points of the bounding box and the lines of the BFQ. The red rectangle and the black dashed quadrilateral are the bounding box and the BFQ, respectively. The green lines of the BFQ make the eight intersection points with the red bounding box, as shown by the green circles.

A straight line obtained by extending a line segment of the vehicle BFQ intersects the edge of the bounding box at two points. Thus, there are eight intersection points as the green circles where the BFQ lines (green lines) meet the red bounding box, as shown in Figure 8. In other words, if the two intersection points can be found on the edges of a bounding box, a straight line of the BFQ is obtained by connecting the two intersection points. The remaining lines of the BFQ are found in the same way. As shown in the figure, two adjacent lines of the BFQ intersect at one point, which is the corner of the BFQ. Therefore, the BFQ can be detected by finding the total number of eight intersection points at the edges of the bounding box.

Intersection point detection can be conducted in two ways. The first one is to find the index of the edge with the intersection point and the offset from a start point to the intersection point. The other one is to find only the offsets from the fixed start point.

**Figure 9:**
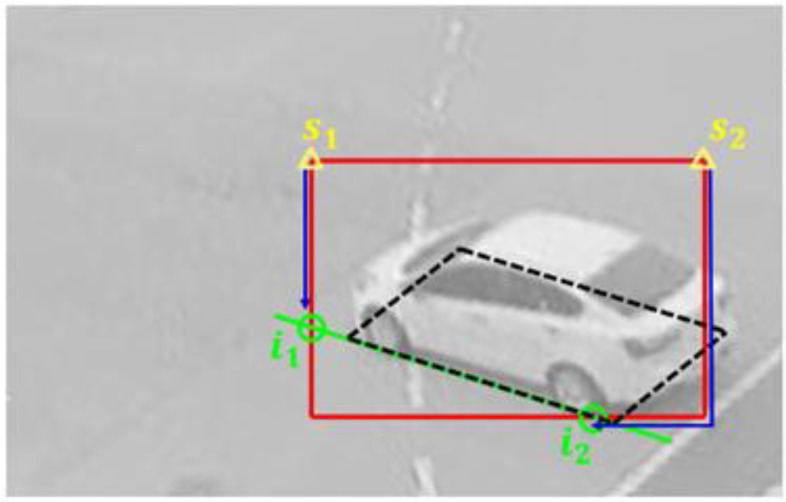
Offset from the corresponding start point to the intersection point. The black dashed quadrilateral indicates the BFQ. The green circles are the intersection points generated by the green line and the red bounding box. The blue arrows depict the offsets from the yellow start points to the corresponding intersection points.

The first one uses different start points depending on the intersection point. If the edge index is given from the network, the start point is determined as the point with the smaller coordinate of the two ends of the edge. In Figure 9, the yellow triangles are the start points $s_{1}$ and $s_{2}$. The intersection points $i_{1}$ and $i_{2}$ are at the left and the bottom edges of the bounding box, respectively. If the indices of these edges are obtained from the network, the smaller ends of each edge become the start points. The offset from the start point to the intersection point is the coordinate difference along the *x*-axis or *y*-axis:

(5) $$offset=\left|s_{k,x}-i_{k,x}\right|+\left|s_{k,y}-i_{k,y}\right|,\;\;\;\;\;k=1,\;\ldots ,\;8,$$

where k is the number of intersection points. The offset is obtained by regressing the ratio $r_{s}$ of the bounding box size W×H and the offset which is calculated with the formula:

(6) $$r_{s}=\frac{\left|s_{k,x}-i_{k,x}\right|}{W}+\frac{\left|s_{k,y}-i_{k,y}\right|}{H},\;\;\;\;\;k=1,\;\ldots ,\;8$$

and the edge index is obtained by four-class classification. The output of the added extra heads is eight sets of the edge index and the ratio values. This is referred to as L2 in the following.

**Figure 10:**
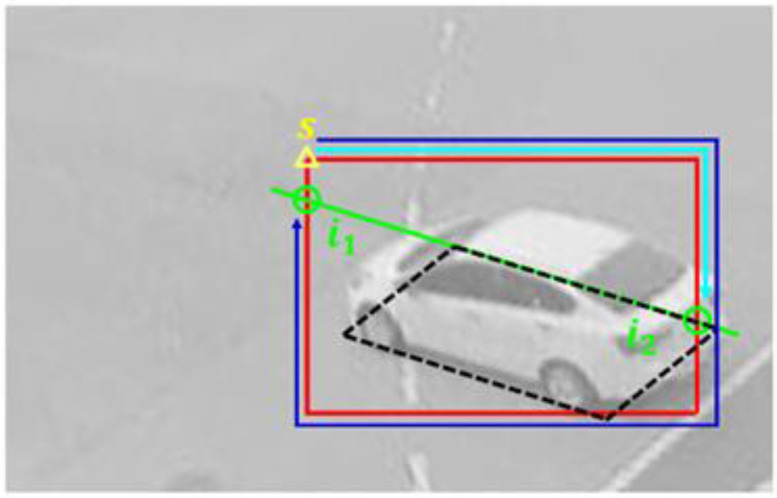
Offset from the fixed start point to the intersection point. The yellow triangle indicates the start point at the top left corner of the bounding box. The green circles are the intersection points of the green line of the BFQ and the red bounding box. The offsets from the start point to the intersection points $i_{1}$ and $i_{2}$ are indicated by the blue and light blue lines, respectively.

Another way to find an offset is to use the fixed start point as the top left corner of the bounding box, as shown in Figure 10. In this figure, the blue and the light blue lines are the offsets from the start point (yellow triangle) to the intersection points (green circles), respectively. Since the start point is only one, the edge index is not needed; only the offsets are needed. The offset is obtained by regressing the ratio of the offset to the perimeter of the bounding box and the ratio is calculated with the formula:

(7) $$r_{s}=\frac{\left|s-i_{k,x}\right|+|s-i_{k,y}|}{2(W+H)},\;\;\;\;\;k=1,\ldots ,\;8$$

Thus, the added extra heads output eight ratio values. This is referred to as L3 in the following.

## 4. Experiments

### 4.1. Dataset and Training

**Figure 11:**
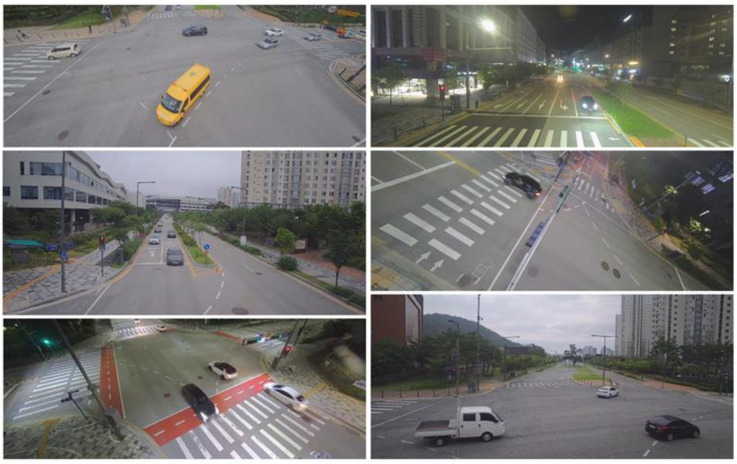
Example images used in the experiment.

To train and test the presented methods, images captured by surveillance cameras were used. Figure 11 shows example images. It can be noticed that cameras were located at various places with different angles. The images were acquired under various lighting conditions and included multiple types of vehicles, such as sedans, vans, trucks, and buses. The training data include 6591 images with 17,249 vehicles, and the test data consist of 1622 images with 4235 vehicles. Vehicle BFQ labels were manually designated by using camera calibration results.

All networks were optimized using an ADAM optimizer, whose learning rate starts at 10^−5^ and reduces using a cosine annealing schedule. Each network was trained for 200 epochs, and the batch size was set to 16. All the experiments were conducted using TensorFlow and NVIDIA TITAN RTX.

### 4.2. Evaluation Criteria

**Figure 12:**
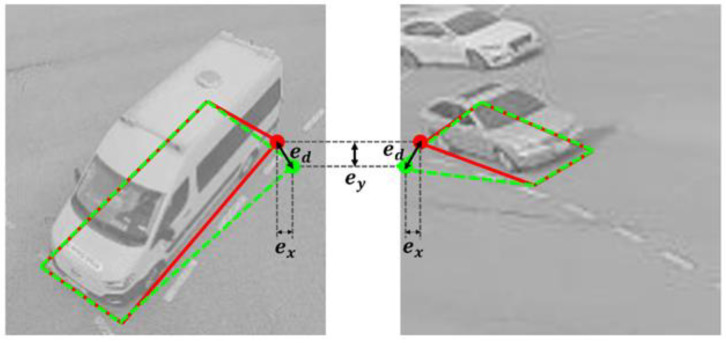
The Euclidean errors affected by the vehicle size in the image. The red and green BFQs are the ground truths and the predictions, respectively. $e_{x}$ and $e_{y}$ are the Euclidean errors along the *x*- and *y*-axis, respectively. $e_{d}$ is the Euclidean error for a corner of the BFQ.

For the evaluation of the BFQ detection accuracy, we relied on the Euclidean distance between the ground truth corners and the detected BFQ corners. However, it’s worth noting that the vehicle size in the image can vary based on their real-world dimensions or their distance from the camera. For example, Figure 12 shows that the Euclidean errors in one corner of the BFQ remains the same despite the different sizes of the vehicles. The ground truth is represented in red, while the predictions are in green. The Euclidean error of a corner, $e_{d}$, can be calculated using:

(8) $$e_{d}=\sqrt{{e_{x}}^{2}+{e_{y}}^{2}},$$

where $e_{x}$ and $e_{y}$ are the Euclidean errors along the *x*-axis and *y*-axis, respectively.

Although $e_{d}$ is the same due to the same $e_{x}$ and $e_{y}$ in Figure 12, the detection classification results need to be different. In this figure, two BFQs with non-perfect shapes are detected. On the left image, the difference between the ground truth BFQ and the detected BFQ is not serious. Conversely, on the right image, this difference is considered as being significant because the vehicle size in the image is smaller. Therefore, only the BFQ on the left image should be classified as correctly detected. To overcome this problem, we normalize the Euclidean errors using the vehicle size. The average of the normalized Euclidean errors of the four corners indicates the BFQ detection accuracy of one vehicle and it is referred to as the position error. The calculation of the average position error over the whole dataset is given by:

(9) $$e=\frac{1}{N}\sum_{i=1}^{N}\frac{1}{4}\sum_{j=1}^{4}\sqrt{{\left(\frac{x_{ij}-{\hat{x}}_{ij}}{W}\right)}^{2}+{\left(\frac{y_{ij}-{\hat{y}}_{ij}}{H}\right)}^{2}},$$

where N is the total number of vehicles. $\left(x_{ij},y_{ij}\right)$ and $\left({\hat{x}}_{ij},{\hat{y}}_{ij}\right)$ are the coordinates of the ground truth corner and the predicted corner, respectively. e is the average position error when the bounding box size of the detected vehicle is W×H. This means the error of the bounding box normalized to 1×1 pixels.

In addition to the Euclidean distance-based measure, the F1 score is also used to evaluate the BFQ detection performance. The F1 score takes into account the normalized error to determine whether a prediction is correct and combines precision and recall using Equation (10), where precision and recall are computed by Equations (11) and (12), respectively. To be considered a correctly detected BFQ, the average position error must be less than the threshold.

(10) $$F_{1}score=2\times \frac{precision\times recall}{precision+recall}$$

(11) $$precision=\frac{the\;number\;of\;correctly\;detected\;bottom\;faces}{the\;number\;of\;detected\;bottom\;faces}$$

(12) $$recall=\frac{the\;number\;of\;correctly\;detected\;bottom\;faces}{the\;number\;of\;ground\;truth\;bottom\;faces}$$

### 4.3. Evaluation Results

Table 3 shows the average precision (AP) of the vehicle bounding boxes detected by each method. As shown in the table, all methods showed similar levels of bounding box AP between 87% and 91%.

Table 4 shows the F1 score, precision, recall, and the average position error evaluated by each method. The F1 score, precision, and recall indicate how many BFQs are detected, and the average position error indicates how accurately they are detected. The final decision for detection is based on two thresholds: 0.2 for strict detection and 0.3 for loose detection. Regardless of the threshold, the L3 method of the line-based approach has the smallest average position error. The C2 of the corner-based approach is in second place.

As seen in Table 4, the methods belonging to the same approach show similar performance. However, there is a notable difference in the performance of each approach. The corner-based approach has small average position errors and, at the same time, high F1 scores. The F1 scores of the PSA-based approach are comparable to those of the corner-based approach, but the average position errors are the largest of the three approaches. Finally, L1 and L2 of the line-based approach have lower F1 scores than the other two approaches but have smaller errors than the PSA-based approach. While L3 provides the smallest error of all the methods, it had the lowest F1 score.

### 4.4. Result Analysis

**Figure 13:**
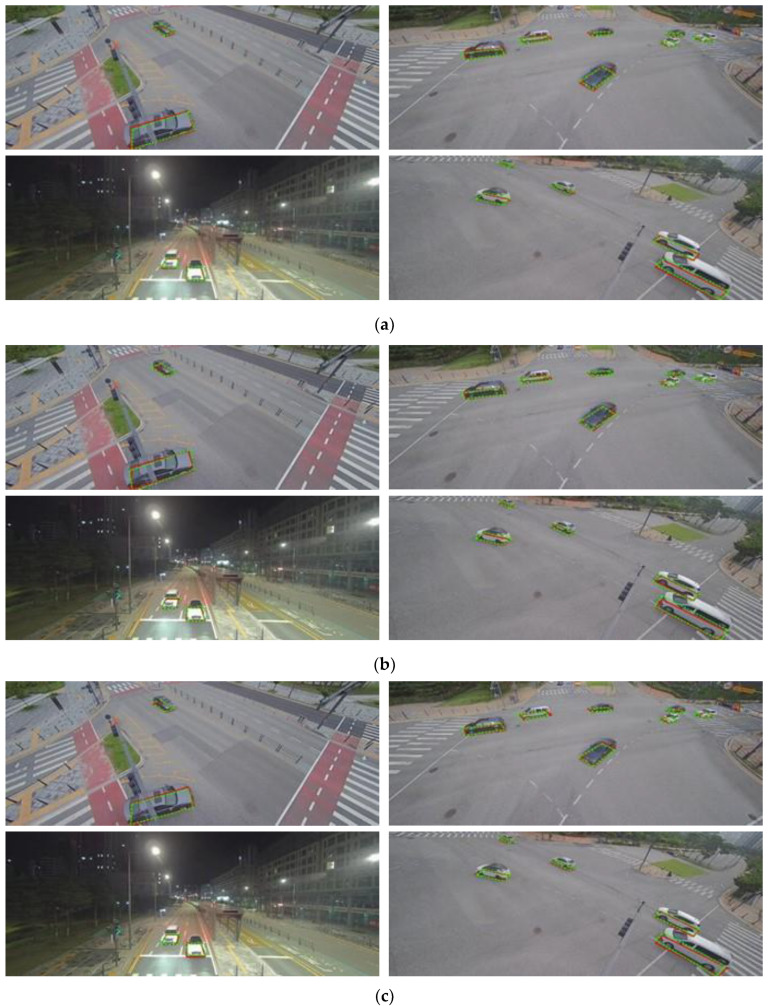
Detection results of the representative methods selected for each approach. The red and the green dashed quadrilaterals represent the ground truth and the detected BFQs, respectively: (**a**) the detected BFQs using the C2 method; (**b**) the detected BFQs using the PSA2 method; and (**c**) the detected BFQs using the L2 method.

Figure 13 depicts the detection results of each approach, illustrating the remarkable differences in their performance. To provide a clear comparison, one representative method was selected for each approach, and the detection results of the selected method are shown in this figure. The red and green dashed quadrilaterals represent the ground truth and the detected BFQs by each approach. The images were taken at different times and locations, mostly on roads with intersections and varying driving directions. Despite these differences, the vehicle BFQs were successfully detected in each case. Furthermore, as shown in the right column of Figure 13, the detections were successful even when the vehicle was far away and appeared small.

**Figure 14:**
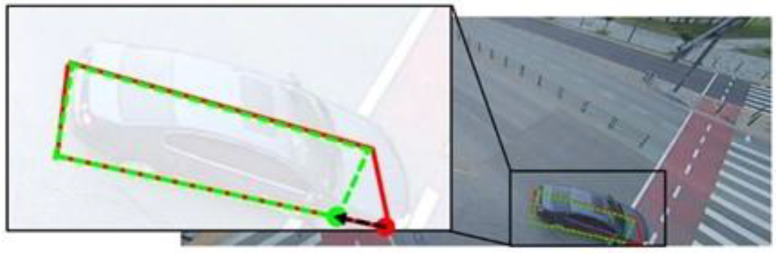
The detected BFQ having a significant error at one corner when using a corner-based approach. The red solid lines and the green dashed lines represent the ground truth and the detected BFQs, respectively. To effectively show the difference between the ground truth and the detected corners, it is indicated by the arrow.

However, the approaches were not always successful in detecting BFQs, as demonstrated in Figure 14. Figure 14 shows a case where a corner-based approach detects the BFQ with the non-perfect shape due to a significant error at one corner. This approach identifies BFQs by detecting four corners, so a failure to detect even one corner can result in a significant difference between the detected BFQ and the actual one, or worse, the detection may fail altogether.

**Figure 15:**
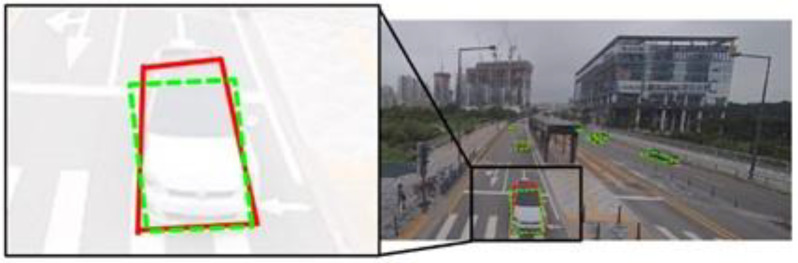
The limitations of the parallelogram assumption of the PSA-based approach. The red and the green dashed quadrilaterals represent the ground truth and the detected BFQs, respectively.

**Figure 16:**
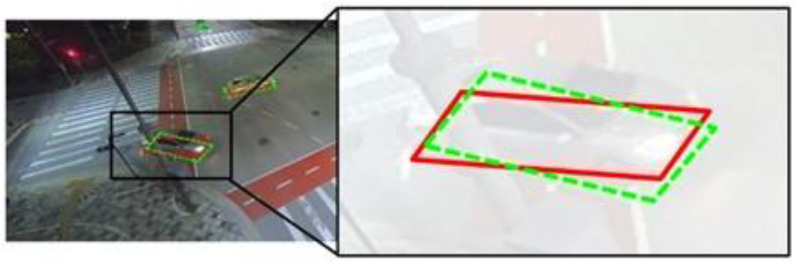
The effect of a single element error on BFQ detection using the PSA-based approach. The red solid lines and the green dashed lines represent the ground truth and the detected BFQs, respectively.

The PSA-based approach identifies the position, the size, and the angle to detect the BFQ, assuming that the BFQ is a parallelogram. However, in the dataset, the BFQ can have different shapes depending on the vehicle’s pose, leading to errors in the parallelogram assumption in most cases. Figure 15 illustrates an example where the ground truth BFQ (red) looks more similar to a rhombus than a parallelogram. Nevertheless, the detected BFQ (green dashed) is a parallelogram due to the assumption. This is an example of the limitations of the parallelogram assumption, which is dependent on the camera angle. In Figure 16, there is a case where the PSA-based approach fails to detect one of the three elements. Although the position and the size are accurately detected, the detected BFQ shows a large difference from the actual one due to the error in the detected angle. This can lead to incorrect vehicle direction estimation based on the shape of the BFQ.

**Figure 17:**
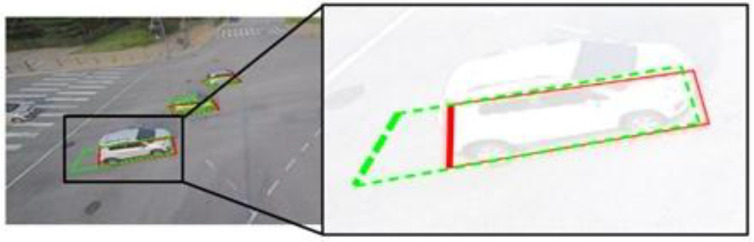
The effect of the small error of a single element when the L1 method is used to detect the BFQ. The red solid and the green dashed quadrilaterals represent the ground truth and the detected BFQs, respectively.

**Figure 18:**
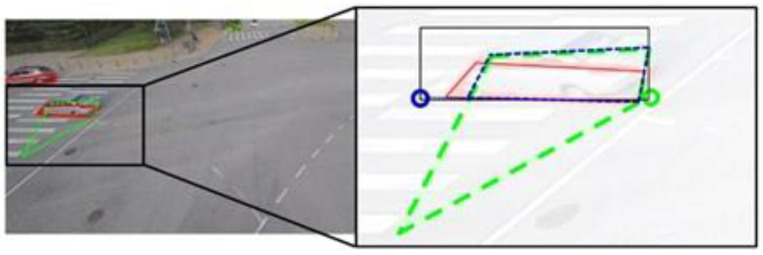
The effect of the misidentified edge index when the L2 method is used to detect the BFQ. The red quadrilateral is the ground truth BFQ, and the black rectangle is the bounding box. The green dashed quadrilateral indicates the BFQ resulting from the intersection point (green circle) where the edge index is misidentified. The blue dashed quadrilateral indicates the BFQ resulting from the intersection point (blue circle) assumed that the edge index is correctly identified.

The line-based approach detects the BFQ by finding its four straight lines. The L1 method of this approach identifies the four sets of the line parameters (cos⁡θ,sin⁡θ,ρ) for a single vehicle. Figure 17 shows the result when one of the 12 parameters fails to be detected. As shown in this figure, a small error in the sin⁡θ parameter among (cos⁡θ,sin⁡θ,ρ) for the left straight line can cause a significant difference in the detected BFQ from the actual one. The remaining methods of the line-based approach identify the intersection points of the bounding box with the lines of the BFQ. Specifically, the L2 method finds the edge index and the ratio for each intersection point. In Figure 18, the detected BFQ resulting from an intersection point with a misidentified edge index (green circle) differs significantly from the actual BFQ. If the edge index is correctly identified, the intersection point would have been at the blue circle, resulting in the blue dashed BFQ. Although the blue BFQ has an error, it can still be considered a correct detection. In contrast, the green BFQ cannot be considered valid because its average position error exceeds the threshold. The importance of correctly identifying the edge index for each intersection point is illustrated in Figure 18, as it restricts the intersection point’s location in this method.

**Figure 19:**
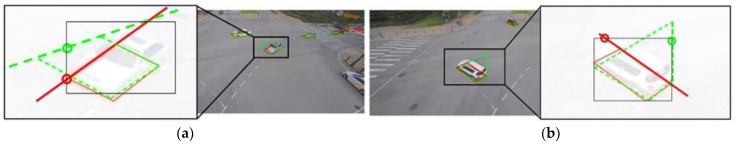
The BFQs resulting from the intersection point with the errors in the predicted ratio. The red circle, line, and quadrilateral are the intersection point, the extended line of an edge, and the BFQ of the ground truth, respectively. The green circle, line, and quadrilateral are the same as the detected BFQ. The bounding box is shown as the black rectangle: (**a**) result of the L2 method; and (**b**) result of the L3 method.

Figure 19 shows the intersection points caused by the ratio prediction errors and the resulting BFQs, indicated by the circles and quadrilaterals. In Figure 19a, the green BFQ results are from the intersection point (green circle) where the edge index is correctly predicted, but the ratio is incorrect. The intersection point location in the L2 method is limited to the width or the height of the bounding box. On the other hand, the L3 method allows the intersection point to be located at any edge of the bounding box. However, this becomes a limitation for intersection points near the corners of the bounding box, as shown in Figure 19b. The predicted intersection point (green circle) in this figure is near the top right corner of the black bounding box. It should be at the top edge, but it is located at the right edge due to an error in the ratio. This illustrates that the creation of a quadrilateral is easily affected by errors in the predicted ratio. The L3 method has many false detections and a low F1 score. However, the results with significant errors are filtered out, resulting in increased accuracy.

## 5. Conclusions

This paper presents a guideline for accurately detecting the vehicle BFQ in surveillance environments. The proposed method involves implementing BFQ detectors by adding extra heads to YOLO and evaluating three approaches: corner-based, PSA-based, and line-based. The corner-based, PSA-based, and line-based approaches encode the BFQ into four corners, a combination of position/size/angle, and four lines, respectively. The quantitative evaluation shows that the corner-based approach has relatively low errors and high detection rates because the position errors of the BFQ corners have less impact on the overall shape of the BFQ compared to the other approaches. On the other hand, the PSA-based approach showed limitations with potential position errors due to the parallelogram assumption of the BFQ, and the line-based approach showed low detection rates because line estimation errors heavily affect the shapes of the quadrilaterals. Since this paper suggests an approach that detects the BFQ for vehicle position estimation by simply adding extra heads to the existing object detector, it is expected to be useful for those needing practical and accurate vehicle detection systems. In the future, we plan to optimize the proposed network by using channel pruning and quantization-aware training to apply it to a real-time embedded system with a neural processing unit (NPU).

## Figures and Tables

**Table 1:** Four methods of the corner-based approach.

Abbreviation of Variations	Encoding Targets	
	Origin	Offset
C1	center of the anchor	ratio to the anchor size
C2	center of the anchor	residual to the anchor size
C3	bottom-left corner of the anchor	ratio to the anchor size
C4	bottom-left corner of the anchor	residual to the anchor size

**Table 2:** Four methods of the PSA-based approach.

Abbreviation of Variations	Encoding Targets		
	Position	Size	Angle
PSA1	C2 applied	residual to the anchor size	two angles
PSA2	C2 applied	residual to the anchor size	cosine and sine of two angles
PSA3	C2 applied	log-scale offset	two angles
PSA4	C2 applied	log-scale offset	cosine and sine of two angles

**Table 3:** Vehicle AP comparison of all BFQ detection methods.

Approach	Method	Vehicle Bounding Box AP (%)
Corner-based	C1	89.42
	C2	88.36
	C3	89.46
	C4	89.34
PSA-based	PSA1	90.28
	PSA2	90.63
	PSA3	90.11
	PSA4	90.36
Line-based	L1	87.98
	L2	87.45
	L3	87.66

**Table 4:** Comparison of BFQ detection results. The detection rate was evaluated through the F1 score, precision, and recall, and detection accuracy was evaluated through the average position error.

Approach	Method	Vehicle BFQ Detection							
		Using Strict Threshold				Using Loose Threshold			
		F1 Score	Precision	Recall	Average Position Error	F1 Score	Precision	Recall	Average Position Error
Corner-based	C1	0.84	0.88	0.81	0.0521	0.92	0.96	0.88	0.0641
	C2	0.86	0.90	0.83	0.0509	0.92	0.96	0.89	0.0614
	C3	0.85	0.88	0.81	0.0515	0.92	0.96	0.89	0.0641
	C4	0.86	0.90	0.83	0.0513	0.92	0.96	0.89	0.0617
PSA-based	PSA1	0.82	0.85	0.78	0.0602	0.89	0.93	0.86	0.0727
	PSA2	0.84	0.88	0.80	0.0605	0.91	0.95	0.87	0.0726
	PSA3	0.82	0.86	0.78	0.0604	0.90	0.95	0.86	0.0739
	PSA4	0.83	0.87	0.79	0.0615	0.91	0.95	0.87	0.0744
Line-based	L1	0.73	0.77	0.68	0.0533	0.83	0.88	0.78	0.0703
	L2	0.75	0.80	0.70	0.0526	0.83	0.89	0.79	0.0670
	L3	0.50	0.54	0.48	0.0389	0.63	0.67	0.60	0.0586

## Data Availability

Data sharing is not applicable to this article.
